# Longitudinal Change in Bone Density, Geometry, and Estimated Bone Strength in Older Men and Women From The Gambia: Findings From the Gambian Bone and Muscle Aging Study (GamBAS)

**DOI:** 10.1002/jbmr.4727

**Published:** 2022-11-11

**Authors:** Mícheál Ó Breasail, Camille Pearse, Ayse Zengin, Landing Jarjou, Cyrus Cooper, Peter R Ebeling, Ann Prentice, Kate A Ward

**Affiliations:** ^1^ Population Health Sciences, Bristol Medical School University of Bristol Bristol UK; ^2^ MRC Nutrition and Bone Health Research Group University of Cambridge Cambridge UK; ^3^ MRC Lifecourse Epidemiology Centre University of Southampton, Southampton General Hospital Southampton UK; ^4^ Department of Medicine, School of Clinical Sciences, Faculty of Medicine, Nursing and Health Sciences, Monash Medical Centre Monash University Clayton Australia; ^5^ MRC Unit The Gambia at London School of Hygiene and Tropical Medicine Banjul Gambia; ^6^ National Institute for Health Research (NIHR) Musculoskeletal Biomedical Research Unit University of Oxford Oxford UK

**Keywords:** AGING, ANALYSIS/QUANTITATION OF BONE, DXA, BONE QCT/μCT, BONE MODELING AND REMODELING, BIOCHEMICAL MARKERS OF BONE TURNOVER

## Abstract

Musculoskeletal aging in the most resource‐limited countries has not been quantified, and longitudinal data are urgently needed to inform policy. The aim of this prospective study was to describe musculoskeletal aging in Gambian adults. A total of 488 participants were recruited stratified by sex and 5‐year age band (aged 40 years and older); 386 attended follow‐up 1.7 years later. Outcomes were dual‐energy X‐ray absorptiometry (DXA) (*n* = 383) total hip areal bone mineral density (aBMD), bone mineral content (BMC), bone area (BA); peripheral quantitative computed tomography (pQCT) diaphyseal and epiphyseal radius and tibia (*n* = 313) total volumetric BMD (vBMD), trabecular vBMD, estimated bone strength indices (BSIc), cross‐sectional area (CSA), BMC, and cortical vBMD. Mean annualized percentage change in bone outcomes was assessed in 10‐year age bands and linear trends for age assessed. Bone turnover markers, parathyroid hormone (PTH), and 25‐hydroxyvitamin D (25(OH)D) were explored as predictors of change in bone. Bone loss was observed at all sites, with an annual loss of total hip aBMD of 1.2% in women after age 50 years and in men at age 70 years plus. Greater loss in vBMD and BSIc was found at the radius in both men and women; strength was reduced by 4% per year in women and 3% per year in men (*p* trend 0.02, 0.03, respectively). At cortical sites, reductions in BMC, CSA, and vBMD were observed, being greatest in BMC in women, between 1.4% and 2.0% per annum. Higher CTX and PINP predicted greater loss of trabecular vBMD in women and BMC in men at the radius, and higher 25(OH)D with less loss of tibial trabecular vBMD and CSA in women. The magnitude of bone loss was like those reported in countries where fragility fracture rates are much higher. Given the predicted rise in fracture rates in resource‐poor countries such as The Gambia, these data provide important insights into musculoskeletal health in this population. © 2022 The Authors. *Journal of Bone and Mineral Research* published by Wiley Periodicals LLC on behalf of American Society for Bone and Mineral Research (ASBMR).

## Introduction

The number of adults aged ≥60 years in Sub‐Saharan Africa (SSA) is currently twice that of northern Europe, a figure that is expected to increase from 46 million in 2015 to 157 million by 2050.^(^ ^1^ ^)^ These demographic changes are occurring alongside rapid urbanization across the region, and together these are increasing the burden of non‐communicable diseases of aging, including osteoporosis and associated fragility fractures.^(^ ^2^ ^)^ Global data on adult hip fractures suggest that the incidence in Africa and Asia is considerably lower than in age‐matched White populations.^(^ ^3^, ^4^, ^5^ ^)^ However, it is predicted that the incidence of hip fracture will increase sixfold to about 6 million in Africa and Asia by 2050.^(^ ^6^ ^)^ This was evidenced recently in a study from South Africa, with incidence rates being much higher than previously reported.^(^ ^7^ ^)^ This will dramatically increase the burden of disease in these countries, with concomitant increases in debilitating morbidity and health care costs.

Despite the predicted rise in fractures, musculoskeletal aging in African populations remains poorly defined,^(^ ^8^ ^)^ and drawing a comparison to aging in the African American population requires caution.^(^ ^9^ ^)^ The evidence base for understanding musculoskeletal health in SSA has to date mostly been drawn from cross‐sectional studies, with few exceptions.^(^ ^10^ ^)^ Our studies of older people in rural Gambia have shown that they have extremely low calcium intakes, low bone mineral density (BMD), and high plasma parathyroid hormone concentrations (PTH), all of which are risk factors for fragility fracture in high‐income populations.^(^ ^11^, ^12^ ^)^ Also, women have high parity after repeated cycles of pregnancy and lactation, which may affect their bone health. We previously studied bone mineral content (BMC) and BMD, measured by single (radius) and dual photon (hip, spine) absorptiometry, in a cross‐sectional study of Gambian women aged older than 44 years and found evidence of bone loss with age, as in high‐income populations.^(^ ^11^ ^)^ More recently, we have published cross‐sectional data from older Gambian men and women using dual‐energy X‐ray absorptiometry (DXA) and peripheral quantitative computed tomography (pQCT).^(^ ^13^ ^)^ However, cross‐sectional studies may over‐ or underestimate bone loss when compared with longitudinal measures,^(^ ^14^, ^15^ ^)^ and to date no studies have documented longitudinal age‐related changes in both men and women beyond midlife.

The aim of this study was therefore to describe annualized changes in BMD, bone geometry, and estimates of strength in Gambian women and men aged ≥40 years to determine whether markers of bone turnover, 25‐hydroxyvitamin D (25(OH)D), and PTH predicted change.

## Materials and Methods

### Recruitment

The study protocol has been published,^(^ ^13^ ^)^ but in brief, we recruited men and women aged ≥40 years, who were identified using the Kiang West Demographic Surveillance System (KWDSS).^(^ ^16^ ^)^ After initial village sensitization and discussion with the elders, participants were located and approached by members of the research team who explained the study in the local language and invited them to participate. The target sample size was 240 women and 240 men, 480 participants in total. Follow‐up measurements were randomized to 1.5 to 2.0 years after the baseline measurement. Participants were recruited and stratified by sex and by 5‐year age bands to ensure equal distribution of participants. Before enrollment, participants were confirmed not to be part of any other ongoing study at MRC Keneba or elsewhere. Pregnant and lactating women were excluded. A woman was considered nonpregnant, nonlactating if she was at least 3 months post lactation and had regular menses. Individuals who were deemed too physically frail or incapable of attending a study visit at MRC Keneba for measurements because of existing disability or chronic illness were excluded from participating at baseline. At follow‐up, for participants who had become too frail to attend the clinic, a home visit was scheduled to collect data on anthropometry (where possible), hand grip strength, and questionnaire.^(^ ^13^ ^)^ All participants gave written, or thumbprint, informed consent. Ethical approval was given by Gambia Government/MRC Unit The Gambia Ethics Committee (SCC#1222).

Sample size was calculated to determine within‐individual change in femoral neck areal bone mineral density (aBMD) as the DXA site with worst coefficient of variation. A sample size of 66 would be needed to detect a 1% change per annum over a 1.75‐year interval with a precision of the estimates (beta) of 30% in the expected rate of change of bone parameters. To detect a 2% change over the same time with 30% precision of the estimates would need a sample size of 16, or 37 for a precision of the estimate 20% expected change. In total hip and pQCT regions, which can be measured with greater coefficient of variation, smaller rates of change would be detectable with this number of participants. As more precise measures than femoral neck (and with fewer missing data because of short femoral neck axis lengths in this population), total hip and pQCT were the outcomes chosen for this article.

### Anthropometry

Baseline height (cm) was measured to the nearest 1 mm using a wall‐mounted stadiometer (Seca GmbH, Hamburg, Germany) and weight (kg) measured to the nearest 0.1 kg using a digital scale (Seca GmbH) while the participants wore light clothing without footwear. Subsequently, body mass index (BMI; kg/m^2^) was calculated.

For pQCT, both forearm and lower‐leg length were measured to the nearest 1 mm using a tape measure: tibia length was measured from the distal edge of the medial malleolus to the tibial plateau; ulna length was recorded as the distance from the olecranon to the ulnar styloid process.

### Bone imaging

A GE Lunar Prodigy Advance (GE Lunar, Waltham, MA, USA; software version 10.0) was used to acquire baseline and follow‐up scans of the proximal femur. Total hip aBMD, bone mineral content (BMC), and bone area (BA) were measured. *T*‐ and *Z*‐scores were calculated as per ISCD guidelines, using NHANES data for *T*‐score calculations and manufacturer reference for *Z*‐scores.^(^ ^17^ ^)^

pQCT scans were acquired using a Stratec XCT2000 and XCT2000L (Stratec Medizintechnik GmBH, Pforzheim, Germany). The European Forearm Phantom (EFP) was used for cross‐calibration between scanners.^(^ ^18^ ^)^ Scan acquisition parameters were voxel size of 0.5 × 0.5 mm, slice thickness of 2 mm, CT scan speed 30 mm/s, and scout view scan speed 40 mm/s speed. Sites of measurement were at the radius (at 4% and 33% of the limb length proximal to the distal endplate) and tibia (at 4% and 38% of the limb length proximal to the distal endplate). The pQCT scans were processed using the manufacturer's software (Stratec XCT version 6.2). At distal 4% sites, CALCBD analysis (contour mode 1, threshold 180 mg/cm^3^, peel mode 1) was used to calculate total cross‐sectional area (CSA) and total and trabecular volumetric bone mineral density (vBMD). Bone strength index of compression (BSIc) was subsequently calculated as total vBMD^2^ × total CSA. At proximal cortical‐rich sites, CORTBD, separation mode 1, threshold 710 mg/cm^3^ was used to define cortical vBMD and area. Total CSA was defined at proximal sites at a threshold of 280 mg/cm^3^. Scans were qualitatively graded by visual inspection to assess their suitability for longitudinal analysis: scan slices with excessive movement or other artifacts, and scout views that did not match longitudinally were excluded (*n* = 73).

### Scanner quality control and assurance

Quality assurance (QA) and quality control (QC) procedures were as per manufacturer guidelines where phantoms were scanned daily for QA and weekly for QC. These also monitor scanner drift and performance over time. Duplicate scans in 30 Gambian adults were used to determine the precision of repeated measured for DXA and pQCT: DXA total hip precision was 0.7% and for pQCT 0.3% to 1.8% for bone measures at the tibia and 1.1% to 6.4% at the radius.

### Bone turnover markers (BTM)

Blood samples were collected in lithium heparin (LH) and EDTA blood tubes from a forearm vein in the morning after an overnight fast. Plasma was separated by centrifugation at 1800*g* for 10 minutes at 4°C, stored at −80°C, and subsequently transported for analysis to the MRC Elsie Widdowson Laboratory (Cambridge, UK) on dry ice and stored at −80°C. EDTA plasma was used for analysis of parathyroid hormone (PTH) and LH serum for bone turnover markers (procollagen type I N‐terminal propeptide [PINP] and serum collagen type 1 crosslinked β‐C‐telopeptide [β‐CTx]) and vitamin D [25(OH)D]. Commercially available assay kits and platforms were used as follows for plasma: plasma intact PTH, β‐CTX, and PINP were measured on the iSys platform (Immunodiagnostics Systems Ltd, Tyne and Wear, UK). For internal plasma drift control: NEQAS (Edinburgh, UK) was used for PTH and NEQAS IIA EQA (Sheffield, UK) for β‐CTX and PINP. 25(OH)D was analyzed in LH plasma using DiaSorin chemiluminescent immunoassay (Liaison; DiaSorin Inc, Stillwater, MN, USA) on an automated analyzer. Assay performance was monitored using kit and in‐house controls and by participation in the Vitamin D External Quality Assessment Scheme (www.deqas.org). All assays performed well and were within specification.

## Statistical analysis

Data analysis was by STATA 15 (StataCorp, College Station, TX, USA). All analyses were conducted in men and women separately as per the original study design and due to the known differences in bone aging between men and women. Between‐visit percentage change was calculated for all pQCT and DXA bone variables by subtracting baseline values from follow‐up, then dividing by baseline value; change was then annualized to allow cross‐cohort comparison. Mean annualized percentage change in bone outcomes is presented in 10‐year age bands by sex and linear trends across age‐band assessed. Linear regression adjusting for baseline bone value was our primary analysis model and used to investigate the associations between age band and DXA and pQCT bone measures. Models were then adjusted for age and height and season of measurement (defined as harvest: January to June; hungry: July to December). Because all adjustments made little difference to findings, we present here only conditional analyses, ie, adjusted for baseline bone.

Bone turnovers markers (β‐CTx, PINP), PTH, and 25(OH)D measurements were transformed using a Fisher‐Yates *Z*‐score transformation to allow interpretation against each other on the same scale. To determine whether these significantly predicted annualized between‐visit change, multiple regression models were constructed to assess each predictor against bone outcome separately, ie, annualized change in each DXA and pQCT variables was regressed by each potential predictor in a separate model. For each outcome of interest, models were (i) adjusted for their respective baseline DXA/pQCT value, (ii) adjusted for baseline bone value and age, (iii) adjusted for baseline bone value, age, and weight, and (iv) adjusted for baseline bone value, age, and height.

Beta coefficients (95% confidence intervals [CI]) from these models reflect the extent to which each independent variable predicts annualized change in bone measures.

As a sensitivity analysis, analyses were repeated in only those with follow‐up pQCT data and results did not differ (data not presented).

## Results

Of the 488 participants who attended baseline visits, 383 Gambian adults (54.4% women) aged 40 to 92 years had repeat DXA scans and 313 had repeat pQCT scans with a median of 1.7 (interquartile range 1.6–1.9) years between scans. Population baseline descriptive data for participants with longitudinal scan data are summarized in Table 1. Mean (SD) baseline *T*‐scores (calculated using NHANES III database) were −1.72 (1.19) in women and −0.17 (0.19) in men.

**Table 1:** Population Descriptives for Men and Women With Longitudinal DXA or pQCT Scans

	Men (*n* = 176)	Women (*n* = 210)
Weight (kg)	60.1 (10.4)	54.6 (9.9)
Height (cm)	169.3 (6.9)	157.8 (6.0)
BMI (kg/m^2^)	20.9 (3.0)	21.9 (3.5)
β‐CTX (ng/mL)	0.72 (0.31)	0.66 (0.29)
PTH (pg/mL)^a^	69.0 [53.1–90.5]	74.4 [55.2–98.6]
PINP^a^ (μg/L)	77.9 [62.2–103.8]	90.8 [65.9–114.5]
25(OH)D (nmol/L)	64.2 (18.1)	68.6 (18.3)
Age band (years)	*n* (%)	*n* (%)
40–49	46 (26.1)	43 (20.5)
50–59	48 (27.3)	60 (28.6)
60–69	36 (20.5)	59 (28.1)
70+	46 (26.1)	48 (22.9)
Season	*n* (%)	*n* (%)
Harvest	109 (61.9)	128 (61.0)
Hungry	67 (38.1)	82 (39.0)

DXA = dual‐energy X‐ray absorptiometry; pQCT = peripheral quantitative computed tomography; BMI = body mass index; β‐CTX = beta CrossLaps; PTH = parathyroid hormone; PINP = procollagen type I N propeptide.

Data are presented as mean (SD).

Harvest season is from January to June and hungry season from July to December.

^a^ Median (interquartile range).

### Annualized change in DXA bone measures in men and women

**Fig. 1:**
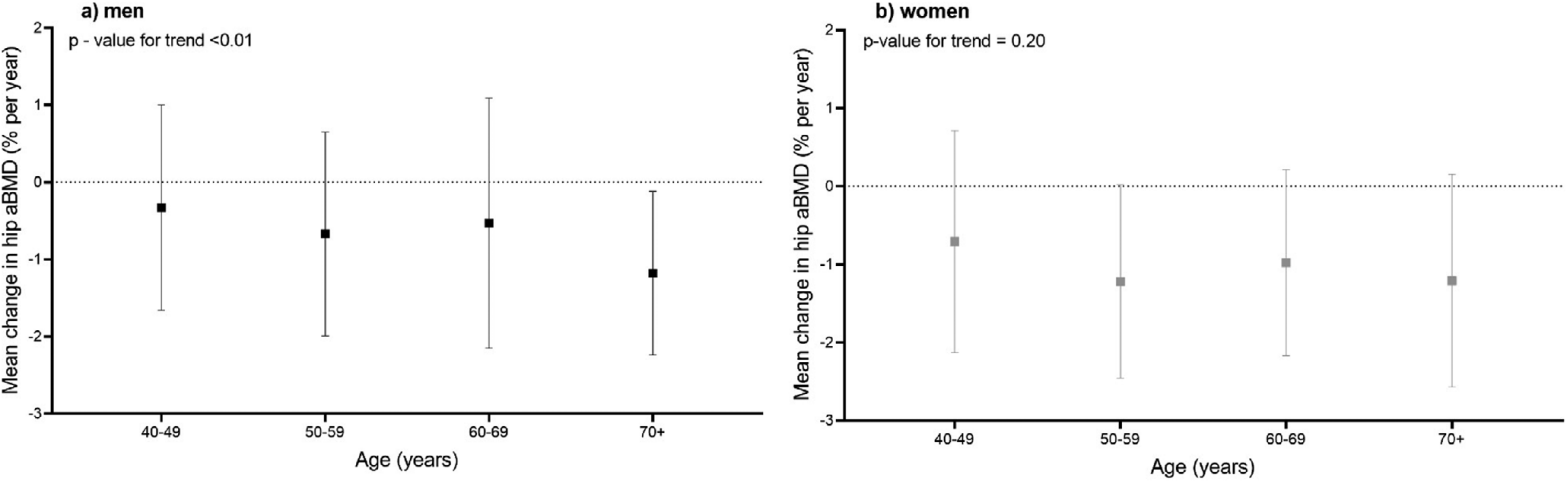
Mean annualized percentage change in total hip areal bone mineral density (aBMD) from dual‐energy X‐ray absorptiometry (DXA) scans in men (*A*) and women (*B*) by 10‐year age bands, calculated from the prospective measurements.

Mean yearly percentage change in total hip aBMD from DXA scans by 10‐year age bands are presented in Fig. 1 and Table 2. The greatest mean bone loss at the total hip site was 1.15% in both men and women across all 10‐year age bands (Fig. 1). The greatest decreases in women were in the years around the menopausal transition (50–59 years), where there were losses more than 1% aBMD per annum (mean [SD] loss 50–59 years was −1.22 [1.24]%, Fig. 1); bone loss continued at 0.98 (1.19)% for decades 60–69 and 1.21 (1.36) 70+ in women (*p* value for trend = 0.2). In men, a similar magnitude of bone loss was found in the 70+ age group (−1.18 [1.06]; *p* value for trend across all age bands <0.01). Bone area increased in men at all ages but not in women; BMC loss followed a similar pattern to BMD.

**Table 2:** Baseline and Annualized Percentage Change, Calculated From the Prospective Measurements, in DXA Bone Measures in Men and Women

	aBMD		BMC		BA	
Age band (years)	Baseline (g/cm^2^)	Annualized % change	Baseline (g)	Annualized % change	Baseline (cm^2^)	Annualized % change
Men						
40–49	1.04 (0.13)	−0.33 (1.33)	34.64 (5.13)	0.11 (1.68)	33.42 (2.47)	0.44 (1.09)
50–59	1.02 (0.10)	−0.67 (1.32)	34.37 (4.15)	−0.21 (1.71)	33.82 (2.16)	0.47 (1.33)
60–69	0.92 (0.12)	−0.53 (1.62)	30.78 (4.30)	−0.29 (1.82)	33.33 (2.06)	0.25 (0.92)
70+	0.92 (0.15)	−1.18 (1.06)	30.72 (5.18)	−1.03 (1.24)	33.46 (2.05)	0.16 (1.04)
Women						
40–49	1.00 (0.13)	−0.71 (1.42)	28.11 (4.66)	−0.82 (1.89)	28.11 (2.11)	−0.12 (1.04)
50–59	0.87 (0.15)	−1.22 (1.24)	24.91 (4.64)	−1.51 (1.73)	28.73 (1.79)	−0.30 (0.98)
60–69	0.77 (0.09)	−0.98 (1.19)	22.29 (3.16)	−1.34 (1.74)	29.01 (2.14)	−0.37 (1.14)
70+	0.70 (0.10)	−1.21 (1.36)	20.01 (4.01)	−1.14 (2.18)	28.52 (2.46)	0.06 (1.40)

Data are presented as mean (SD).

DXA = dual‐energy X‐ray absorptiometry; aBMD = areal bone mineral density; BMC = bone mineral content; BA = bone area.

### Annualized change in pQCT bone parameters in men and women

**Fig. 2:**
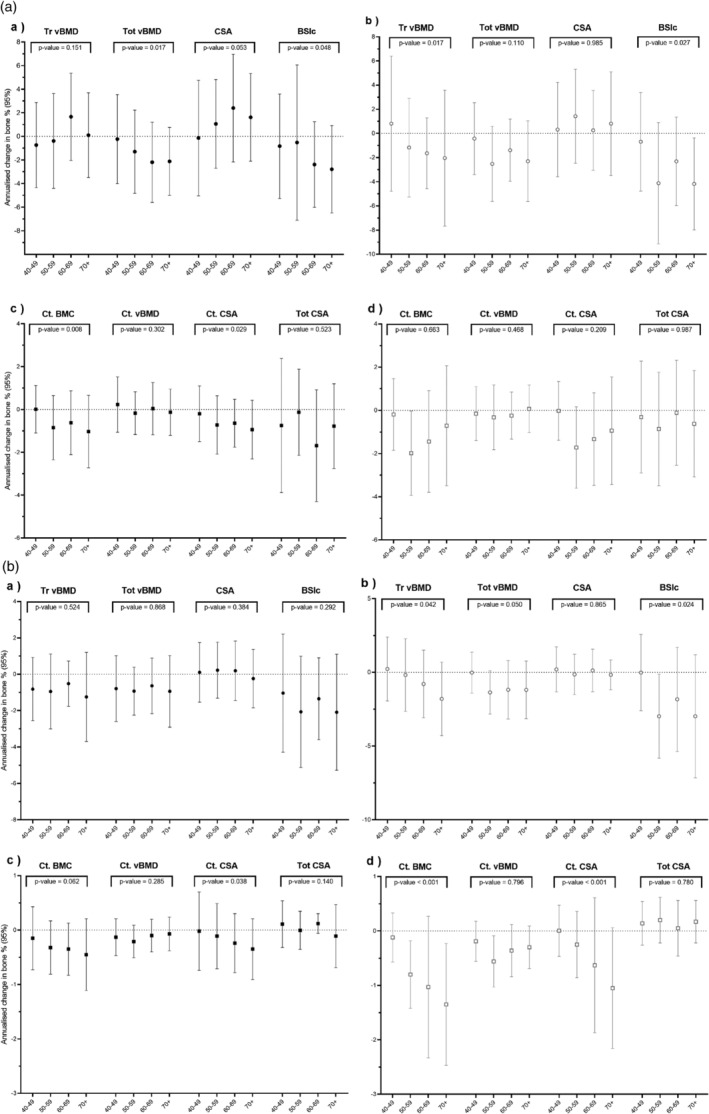
(*A*) Mean (95% confidence interval [CI]) annualized percentage change (calculated from the prospective measurements) in bone measures (*a*) radius 4% men, (*b*) radius 4% women, (*c*) radius 33% men, and (*d*) radius 33% women by 10‐year age bands. (*B*) Mean (95% CI) annualized percentage change (calculated from the prospective measurements) in bone measures (*a*) tibia 4% men, (*b*) tibia 4% women, (*c*) tibia 38% men, and (*d*) tibia 38% women by 10‐year age bands. Tr = trabecular; vBMD = volumetric bone mineral density; Tot = total; CSA = cross‐sectional area; BSIc = bone strength index of compression; Ct = cortical; BMC = bone mineral content.

**Fig. 3:**
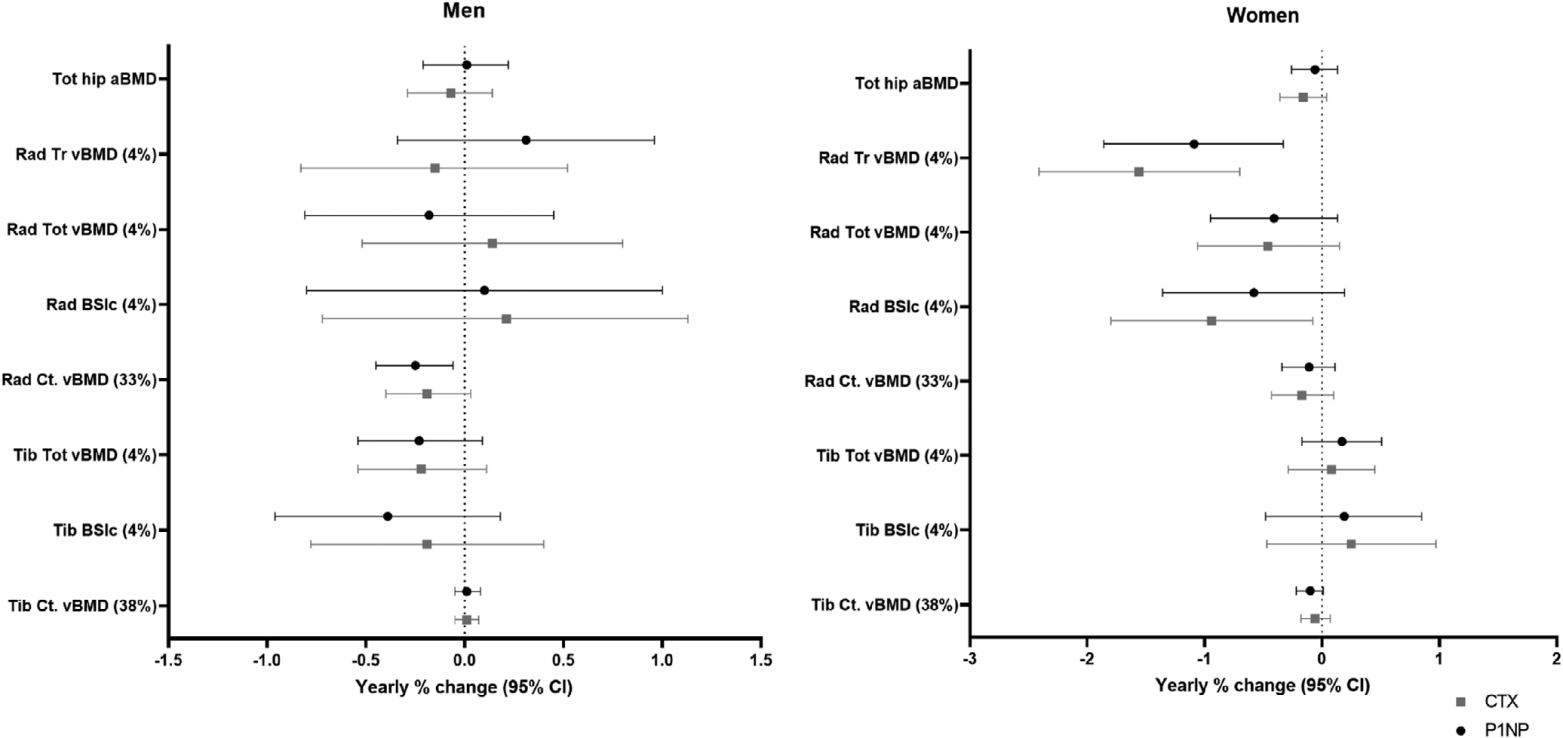
Associations between baseline biomarkers (Fisher‐Yates *Z*‐scores) and yearly percentage change in bone measures (calculated from the prospective measurements), in men and women adjusted for baseline bone value. Changes shown per one SD difference in bone turnover markers. Tot = total; aBMD = areal bone mineral density; Rad = radius; Tr = trabecular; vBMD = volumetric bone mineral density; BSIc = bone strength index of compression; Ct = cortical; Tib = tibia.

Mean yearly percentage change in pQCT bone outcomes at the radius and tibia by 10‐year age bands are shown in Figs. 2*A*, *B* and 3 and Tables 3 and 4. In men, radial total vBMD and BSIc losses were greater at older ages (ie, aged 60 years and older), with respective losses of 2.19 (3.40)% and 2.79 (3.70)% per annum (*p* value for trend = 0.02 and 0.05, respectively). However, CSA increased across age bands (*p* value for trend = 0.05), with the greatest increase (2.40 [4.57]%) found in men aged older than 70 years (Fig. 2*A*). At the 33% (diaphyseal) radius, in men both BMC and cortical CSA decreased within the majority of age bands, the magnitude of which appeared to increase with increasing age (*p* value for trend = 0.01 and 0.03, respectively) (Fig. 2*A*). In women, radius 4% (epiphyseal) trabecular vBMD (−2.3% [5.8%]) and BSIc (up to 4% in those aged 70 years and older) losses were greater with increasing age (*p* value for trend = 0.02 and 0.03, respectively) (Fig. 2*A*). No consistent pattern of change was observed for diaphyseal radius pQCT outcomes in women nor for cortical vBMD or total CSA in men (Fig. 2*A*).

**Table 3:** Baseline and Annualized Percentage Change, Calculated From the Prospective Measurements, in pQCT Radius 4% and Tibia 4% Bone Measures in Men and Women

	Tr vBMD (4%)		Tot vBMD (4%)		CSA (4%)		BSIc (4%)	
Age band (years)	Baseline	Annualized % change	Baseline	Annualized % change	Baseline	Annualized % change	Baseline	Annualized % change
Radius								
Men								
40–49	191.3 (41.8)	−0.74 (3.61)	342.0 (45.4)	−0.23 (3.77)	421.3 (73.9)	−0.14 (4.90)	0.48 (0.11)	−0.83 (4.43)
50–59	176.1 (37.2)	−0.39 (4.02)	328.4 (54.2)	−1.30 (3.53)	401.0 (61.2)	1.06 (3.75)	0.43 (0.13)	−0.52 (6.58)
60–69	145.4 (34.0)	1.66 (3.70)	281.8 (36.2)	−2.19 (3.40)	408.2 (64.3)	2.40 (4.57)	0.32 (0.07)	−2.39 (3.63)
70+	143.7 (34.6)	0.10 (3.59)	280.3 (38.3)	−2.12 (2.88)	399.8 (58.0)	1.61 (3.72)	0.31 (0.07)	−2.79 (3.70)
Women								
40–49	141.0 (36.5)	0.81 (5.60)	300.3 (45.9)	−0.43 (2.98)	330.9 (48.7)	0.32 (3.91)	0.30 (0.07)	−0.69 (4.08)
50–59	120.8 (27.0)	−1.17 (4.09)	258.9 (37.5)	−2.52 (3.11)	337.4 (36.2)	1.42 (3.90)	0.23 (0.06)	−4.12 (5.02)
60–69	170.8 (27.5)	−1.64 (2.93)	233.1 (45.5)	−1.39 (2.57)	353.2 (48.2)	0.26 (3.08)	0.19 (0.07)	−2.31 (3.66)
70+	94.1 (22.8)	−2.04 (5.63)	222.4 (38.0)	−2.30 (3.34)	333.9 (43.5)	0.81 (4.29)	0.17 (0.06)	−4.18 (3.80)
Tibia								
Men								
40–49	188.3 (34.4)	−0.82 (1.74)	292.3 (41.3)	−0.79 (1.81)	1103.7 (121.2)	0.10 (1.64)	0.95 (0.26)	−1.04 (3.25)
50–59	177.7 (25.8)	−0.95 (2.06)	279.1 (37.7)	−0.93 (1.32)	1103.6 (144.2)	0.22 (1.54)	0.86 (0.18)	−2.07 (3.06)
60–69	173.7 (31.6)	−0.52 (1.25)	255.5 (37.7)	−0.64 (1.53)	1139.8 (161.2)	0.19 (1.64)	0.74 (0.17)	−1.35 (2.25)
70+	159.5 (27.8)	−1.25 (2.45)	246.7 (34.8)	−0.94 (1.97)	1090.1 (132.7)	−0.24 (1.61)	0.67 (0.17)	−2.09 (3.19)
Women								
40–49	192.9 (28.4)	0.23 (2.17)	285.0 (35.9)	−0.02 (1.39)	890.1 (102.3)	0.20 (1.53)	0.72 (0.14)	−0.02 (2.59)
50–59	160.2 (36.2)	−1.81 (2.46)	246.7 (37.4)	−1.36 (1.47)	938.0 (125.7)	−0.14 (1.37)	0.57 (0.16)	−2.98 (2.85)
60–69	137.1 (29.1)	−0.79 (2.30)	214.6 (31.2)	−1.18 (1.98)	947.0 (133.0)	0.13 (1.45)	0.44 (0.12)	−1.83 (3.54)
70+	130.4 (27.7)	−1.80 (2.49)	198.5 (28.2)	−1.19 (1.96)	939.4 (134.9)	−0.17 (1.01)	0.37 (0.11)	−2.98 (4.17)

Data are presented as mean (SD).

pQCT = peripheral quantitative computed tomography; Tr = trabecular; vBMD = volumetric bone mineral density; Tot = total; CSA = cross‐sectional area; BSIc = bone strength index of compression.

**Table 4:** Baseline and Annualized Percentage Change, Calculated From the Prospective Measurements, in pQCT Radius 33% and Tibia 38% Bone Measures in Men and Women

	Radius							
	Ct. BMC (33%)		Ct. vBMD (33%)		Ct. CSA (33%)		CSA (33%)	
Age band (years)	Baseline (mg/mm)	Annualized % change	Baseline (mg/cm^3^)	Annualized % change	Baseline (mm^2^)	Annualized % change	Baseline (mm^2^)	Annualized % change
Radius								
Men								
40–49	118.0 (13.0)	0.01 (1.11)	1222.3 (27.9)	0.23 (1.29)	96.5 (10.6)	−0.20 (1.30)	138.7 (19.8)	−0.75 (3.13)
50–59	117.9 (15.9)	−0.85 (1.50)	1222.8 (30.5)	−0.17 (1.00)	96.4 (11.9)	−0.72 (1.36)	140.9 (18.7)	−0.13 (2.01)
60–69	108.0 (13.2)	−0.62 (1.49)	1203.1 (25.1)	0.04 (1.22)	89.7 (10.7)	−0.64 (1.12)	139.0 (20.2)	−1.69 (2.61)
70+	104.7 (16.5)	−1.03 (1.69)	1195.7 (27.3)	−0.13 (1.08)	87.4 (13.0)	−0.94 (1.37)	136.5 (18.5)	−0.78 (1.98)
Women								
40–49	87.1 (11.7)	−0.19 (1.66)	1228.5 (33.5)	−0.15 (1.25)	71.0 (8.8)	−0.02 (1.36)	104.7 (12.2)	−0.31 (2.59)
50–59	78.9 (15.0)	−1.98 (1.95)	1186.8 (49.8)	−0.32 (1.50)	66.4 (10.9)	−1.72 (1.88)	109.6 (17.3)	−0.86 (2.63)
60–69	69.0 (13.8)	−1.44 (2.35)	1153.4 (40.1)	−0.24 (1.09)	59.7 (10.3)	−1.33 (2.14)	108.3 (10.5)	−0.11 (2.43)
70+	63.7 (11.6)	−0.71 (2.78)	1144.2 (28.2)	0.07 (1.10)	55.6 (9.2)	−0.94 (2.49)	110.2 (16.8)	−0.62 (2.46)
Tibia								
Men								
40–49	380.7 (49.9)	−0.15 (0.58)	1216.2 (24.0)	−0.13 (0.34)	312.1 (38.6)	−0.02 (0.72)	461.6 (65.1)	0.11 (0.43)
50–59	379.0 (47.6)	−0.32 (0.49)	1211.5 (29.3)	−0.21 (0.30)	311.7 (36.9)	−0.11 (0.60)	460.7 (51.6)	−0.004 (0.35)
60–69	347.4 (45.0)	−0.35 (0.48)	1199.8 (27.2)	−0.10 (0.30)	288.6 (64.7)	−0.24 (0.54)	446.5 (54.9)	0.12 (0.18)
70+	345.4 (39.1)	−0.45 (0.66)	1196.2 (26.7)	−0.07 (0.31)	288.5 (31.4)	−0.35 (0.56)	442.4 (56.7)	−0.11 (0.58)
Women								
40–49	278.1 (37.6)	−0.12 (0.45)	1218.1 (27.9)	−0.19 (0.37)	227.1 (27.4)	0.005 (0.47)	350.3 (46.0)	0.14 (0.40)
50–59	260.0 (40.8)	−0.80 (0.62)	1176.9 (40.0)	−0.56 (0.47)	219.4 (28.7)	−0.25 (0.61)	361.9 (43.8)	0.20 (0.42)
60–69	240.9 (48.8)	−1.03 (1.30)	1144.9 (54.7)	−0.36 (0.48)	209.0 (37.0)	−0.63 (1.24)	363.6 (48.0)	0.05 (0.51)
70+	209.9 (39.2)	−1.35 (1.12)	1138.1 (43.9)	−0.30 (0.39)	183.6 (29.7)	−1.05 (1.11)	349.0 (50.3)	0.17 (0.39)

pQCT = peripheral quantitative computed tomography; vBMD = volumetric bone mineral density; CSA = cross‐sectional area; Ct = cortical.

Data are presented as mean (SD).

Bone losses of 0.5% to 1.5% were found at the epiphyseal tibia in both men and women, although no consistent trend by age was detected (Fig. 2*B*). Loss of BSIc of 2.98 (4.17)% in women and 2.09 (3.19)% in men were observed. At the 38% tibia, cortical CSA and cortical vBMD decreased by age band (*p* value for trend = 0.04, 0.06 respectively) (Fig. 2*B*). In women at the epiphyseal tibia, trabecular vBMD, total vBMD, and BSIc losses were greater by age band (*p* value for trend = 0.04, 0.05 and 0.02, respectively) (Fig. 2*B*). At 38% tibia, in women, tibia BMC and cortical CSA losses were between 1% and 1.3% per year, increasing by 10‐year age band (*p* value for trend for both <0.01) (Fig. 2*B*).

### Bone turnover markers and bone analytes as predictors of annualized change

Associations between baseline bone turnover markers and yearly percentage change in bone parameters, adjusted for baseline bone value, are presented in Fig. 3. Supplemental Tables S1–S3 detail the impact of further adjustment for age, weight, and height.

No associations between β‐CTX, PINP, PTH, or 25(OH)D were found with change in hip aBMD (Supplemental Table S1).

In women, higher concentrations of plasma β‐CTX and PINP at baseline were negatively associated with subsequent longitudinal changes in trabecular vBMD; similar associations were found for CTX levels and decreasing BSIc at the radius (Fig. 3; Supplemental Table S2). For men, negative associations were observed between baseline PINP and change in both cortical BMC and cortical vBMD. Baseline β‐CTX was only associated with decreasing cortical vBMD. These relationships were robust to adjustment for age with the exception of the association between PINP and BSIc in women, which was attenuated after adjustment (*p* = 0.14).

In women, baseline 25(OH)D levels were positively associated with change in tibial trabecular vBMD and CSA at the epiphysis. Baseline PTH was positively associated with changes in epiphyseal CSA and BSIc at the tibia in men. These associations were all robust to adjustment for age.

Further adjustment for weight or height in addition to baseline bone value and age had little impact on the effect size (Tables 1, 2, 3).

## Discussion

These are the first longitudinal musculoskeletal DXA and pQCT data in older Sub‐Saharan African men and women. In women, as would be expected, the greatest decreases were in those in the years around the menopausal transition, where they lost in excess of 1% aBMD per annum. The magnitude of change was greater in the appendicular skeleton than at the hip in women for most measures; this may be because of better sensitivity of pQCT in detecting age‐related changes in this population. In addition, there were also site‐ and compartment‐specific differences between trabecular and cortical bone in the load‐bearing and non‐load‐bearing limbs. In women, decreases in both total and trabecular vBMD of 1.70% and 1.06% per annum, respectively, were observed at the radius. However, at the load‐bearing epiphyseal tibia, bone loss was mostly the result of decreasing total vBMD, indicating changes in the cortical–subcortical compartment. In men, bone loss was evident through decreases in total vBMD at the epiphyseal radius (1.39%) and tibia (0.85%), with no evidence of trabecular decline. In the cortical compartment, annualized changes were greater in women, though both sexes had greater declines at the radius compared with the tibia. The exception to this was in men, where age‐related expansion of the epiphyseal radius CSA was twofold that in women.

Comparison to other cohorts can be difficult because of differences in technology (DXA versus QCT/pQCT), scan sites (hip versus spine versus appendicular skeleton), and follow‐up periods.^(^ ^19^, ^20^ ^)^ Many of the most relevant longitudinal studies have focused on specific life stages such as the menopause transition or bone changes with advanced age but do not span the complete age range of the present cohort and by design may include participants of a single sex. As such comparisons require caution.

The Framingham Osteoporosis Study found that in predominantly White participants, aged 67 to 90 years old, over an average 4‐year follow‐up period, femoral neck aBMD decreased by 3% to 4% in women, which exceeded age‐associated bone loss in men, with similar patterns at the lumbar spine and forearm.^(^ ^21^ ^)^ Similarly, we observed that the magnitude of bone loss found in women exceeded that of men in the same age band, as evidenced by both DXA and pQCT. At the total hip, we found that there was a significant increase in BA, across our age range, in both sexes (albeit less in women), in keeping with previous research, which suggested compensatory changes in bone geometry during aging.^(^ ^22^, ^23^ ^)^ In men, we observed that the greatest rate of aBMD decline occurred from the age of 70 years onward; this may be important as in other cohorts the rate at which bone is lost has been highlighted as an important risk factor for future fracture, particularly at the hip.^(^ ^24^ ^)^ In the Study of Women's Health Across the Nation (SWAN), a multi‐ethnic US cohort including African American women,^(^ ^25^ ^)^ cumulative 10‐year aBMD loss at the femoral neck was 9.1%, with 5.8% of that loss over menopausal transition in White participants, with a slightly slower rate of loss in African American women. It is difficult to directly compare our data with these from SWAN, but annual total hip aBMD loss in 50‐ to 59‐year‐olds was 1.15%.

Although less widely used than DXA, several cohorts have reported age‐related annualized changes with pQCT. A Belgian study using single‐slice pQCT found postmenopausal women aged 50 to 85 years had annualized vBMD loss of 1.14%, 1.10%, and 0.57% for radius total, trabecular, and cortical vBMD, respectively.^(^ ^26^ ^)^ A Finnish study of pre‐ and postmenopausal women over a 5‐year period reported declines in both bone compartments of the radius and tibia.^(^ ^27^ ^)^ Multi‐slice high‐resolution pQCT (HR‐pQCT) data have also been published, although differing scan sites and measured variables make direct compartment‐specific comparisons difficult. However, comparing our data to that of a large Canadian cohort,^(^ ^15^ ^)^ change in total vBMD in Gambian women exceeded the rate of each 10‐year age band (−1.5% to −2.4% radius, −1.1% to −1.4% tibia) reported in the Canadian cohort (radius −0.3% to −1.3% and −0.4% and 0.9% at the tibia). Similarly, the rate of loss of radius and tibia total vBMD in Gambian men (−1.3% to −2.3% radius, −0.8% to −1.0% tibia) also exceeded that of the Canadian cohort (radius −0.2%, −0.3% to −1.1% at the tibia).^(^ ^15^ ^)^ Similarly Riggs and colleagues reported HR‐QCT measured radius and tibia trabecular vBMD by 10‐year age bands in men and women; losses ranged from −0.2% to 1%, again less than those changes found in our Gambian cohort.^(^ ^28^ ^)^ In the SWAN study, longitudinal HR‐pQCT data showed similar rates of loss in women at the distal radius and tibia to those we observed in Gambian women in the current study.^(^ ^29^ ^)^ Likewise, recent work, in men, by Wagner and colleagues found similar HR‐pQCT decreases in cortical bone and estimated bone strength at tibia accelerated with age.^(^ ^30^ ^)^

### Bone turnover markers

In our study, baseline β‐CTX and PINP predicted change in radius trabecular vBMD in women; these associations were robust to adjustment for age. In men, PINP was negatively associated with diaphyseal radius cortical vBMD and positively associated with CSA. BTMs did not predict change in hip aBMD but were associated with losses at the radius, trabecular vBMD, and BSIc in women and cortical vBMD in men. Only a few studies from SSA have measured BTMs in adulthood^(^ ^31^, ^32^, ^33^ ^)^ and into advanced age^(^ ^34^ ^)^ but did not examine BTMs as predictors of longitudinal change. In population‐based studies in HIC, BTMs modestly predict bone loss in postmenopausal women,^(^ ^28^, ^35^, ^36^ ^)^ and while there is some evidence that BTMs predict bone loss in elderly men,^(^ ^28^ ^)^ others have suggested their clinical utility may be limited.^(^ ^19^ ^)^ Riggs and colleagues report β‐CTX and PINP as significant predictors of bone loss in postmenopausal women and men aged older than 50 years,^(^ ^28^ ^)^ though this differed between trabecular and cortical bone outcomes. A large Icelandic cross‐sectional study of men and women found weak negative associations between BTMs (β‐CTX and PINP) and QCT lumbar spine and femoral neck vBMD in older adults.^(^ ^37^ ^)^ Another cross‐sectional study reported BTMs (PINP and N‐terminal telopeptide of type I collagen) were negatively associated with ultradistal radius bone microarchitecture in both sexes.^(^ ^38^ ^)^ Longitudinal Swedish data in postmenopausal women found those with the highest BTM levels had greater aBMD decreases compared with those with lower levels.^(^ ^39^ ^)^ In the OFELY Study, BTMs were most strongly negatively correlated with forearm aBMD in early postmenopausal women compared with premenopausal and older postmenopausal women.^(^ ^40^ ^)^ BTMs were reported to be associated with bone loss over 7.5 years in men aged ≥50 years and did not predict incident fractures, although statistical power in that study was poor.^(^ ^41^ ^)^

In GamBAS, there were some associations between higher 25(OH)D levels and less bone loss and greater area gain at the tibia in women. In men, higher PTH was associated with less loss of vBMD and less change in CSA and consequently maintenance of bone strength estimates at the tibia. Whether there are interactions with other lifestyle factors explaining these sex differences has not yet been determined, but previously higher PTH has been hypothesized to be protective for bone health in older Gambians.^(^ ^12^ ^)^

### Strengths and limitations

These are the largest, and only, longitudinal aging data to be presented from both men and women in Sub‐Saharan Africa. This cohort has an almost even number of men and women, which are balanced evenly across the decades of older adulthood consistent with original study design. Of the original 488 participants, 23 were lost to follow‐up (4 withdrew consent, 4 died, 9 were lost to follow‐up, 6 were too frail or sick) and 82 were too frail to attend clinic for bone measurements. In addition to presenting annualized change over a 1.7‐year period in both sexes, we have also described predictive value of BTMs on bone. The limitations of this work relate primarily to loss to follow‐up. An important limitation is that we did not have menopause status data to allow us to further explore the impact of the menopause transition on the annualized bone changes we observed in women. Staging menopause by interview/questionnaire is incredibly difficult in this setting as there are appreciable difficulties in translating and communicating some the nuances of menopause into the local Mandinka language. Despite our best efforts, previous attempts to quantify menopause status have been unsuccessful, including exploring the utility of measuring follicle‐stimulating hormone (FSH) within this study population. However, as in other populations, there is wide variation between individuals and the ability to discriminate clearly was limited. Although the direction of associations between PTH and total vBMD, and vitamin D and aBMD were unexpected, we cannot wholly rule out the possibility that these occurred due to chance.

These data provide the first longitudinal evidence of age‐related bone mineral change at axial and appendicular skeletal sites in older men and women living in SSA. With DXA, we observed greater annualized decreases in aBMD at the total hip in women coinciding with age bands where menopause is most likely to occur. Annualized losses are like those found in other populations where there are high osteoporosis and fragility fracture rates. In contrast, men were found to have the highest annualized aBMD reductions with more advanced age. In both men and women, bone loss at the radius was apparent in both trabecular and cortical bone. In women, BTMs predicted loss, independent of age, at the distal radius, a common fragility fracture site in women. The greatest age‐related decreases in both sexes were for estimates of bone strength at the distal radius and tibia, which may be important for fragility fracture etiology. These data provide important insights into musculoskeletal health and bone loss in a resource‐limited population, where fracture rates are predicted to increase exponentially over the coming decades.

## Disclosures

The authors have no conflicts of interest to declare.

## Author Contributions

**Mícheál Ó Breasail:** Data curation; formal analysis; investigation; methodology; writing – original draft; writing – review and editing. **Camille Pearse:** Data curation; formal analysis; investigation; methodology; visualization; writing – original draft; writing – review and editing. **Ayse Zengin:** Data curation; investigation; methodology; writing – original draft; writing – review and editing. **Landing Jarjou:** Investigation; project administration; writing – review and editing. **Cyrus Cooper:** Resources; writing – review and editing. **Peter R Ebeling:** Resources; writing – review and editing. **Ann Prentice:** Conceptualization; funding acquisition; investigation; methodology; resources; supervision; writing – review and editing. **Kate A Ward:** Conceptualization; data curation; formal analysis; funding acquisition; investigation; methodology; project administration; resources; supervision; writing – original draft; writing – review and editing.

### Peer Review

The peer review history for this article is available at https://publons.com/publon/10.1002/jbmr.4727.

## Supporting information


**Supplemental Table S1.** Associations between baseline biomarkers (Fisher‐Yates *Z*‐scores) and annualized percentage change (calculated from the prospective measurements) in DXA total hip measures, in men and women adjusted for: (1) baseline DXA value; (2) baseline DXA value and age; (3) baseline DXA value, age, and weight; (4) baseline DXA value, age, and height.
**Supplemental Table S2.** Associations between baseline biomarkers (Fisher‐Yates *Z*‐scores) and annualized percentage change (calculated from the prospective measurements) in radius pQCT measures, in men and women adjusted for: (1) baseline pQCT value; (2) baseline pQCT value and age; (3) baseline pQCT value, age, and weight; (4) baseline pQCT value, age, and height.
**Supplemental Table S3.** Associations between baseline biomarkers (Fisher‐Yates *Z*‐scores) baseline and annualized percentage change (calculated from the prospective measurements) in tibia pQCT measures, in men and women adjusted for: (1) baseline pQCT value; (2) baseline pQCT value and age; (3) baseline pQCT value, age, and weight; (4) baseline pQCT value, age, and height.

## Data Availability

The data that support the findings of this study are available from the corresponding author upon reasonable request.
