# Supplementary material for: Longitudinal Change in Bone Density, Geometry, and Estimated Bone Strength in Older Men and Women From The Gambia: Findings From the Gambian Bone and Muscle Aging Study (GamBAS)
Source: J Bone Miner Res. 2022 Nov 11;38(1):48–58. doi: 10.1002/jbmr.4727 (PMC10098512; doi:10.1002/jbmr.4727)
Supplement: Supplementary file 1 — Supplemental Table S1. Associations between baseline biomarkers (Fisher‐Yates Z‐scores) and annualized percentage change (calculated from the prospective measurements) in DXA total hip measures, in men and women adjusted for: (1) baseline DXA value; (2) baseline DXA value and age; (3) baseline DXA value, age, and weight; (4) baseline DXA value, age, and height. Supplemental Table S2. Associations between baseline biomarkers (Fisher‐Yates Z‐scores) and annualized percentage change (calculated from the prospective measurements) in radius pQCT measures, in men and women adjusted for: (1) baseline pQCT value; (2) baseline pQCT value and age; (3) baseline pQCT value, age, and weight; (4) baseline pQCT value, age, and height. Supplemental Table S3. Associations between baseline biomarkers (Fisher‐Yates Z‐scores) baseline and annualized percentage change (calculated from the prospective measurements) in tibia pQCT measures, in men and women adjusted for: (1) baseline pQCT value; (2) baseline pQCT value and age; (3) baseline pQCT value, age, and weight; (4) baseline pQCT value, age, and height. [file JBMR-38-48-s001.docx]

Supplementary Table 1. Associations between baseline biomarkers (Fisher-Yates Z scores) and annualized yearly percentage change (calculated from the prospective measurements) in DXA total hip measures, in men and women adjusted for: (1) baseline DXA value; (2) baseline DXA value and age; (3) baseline DXA value, age and weight; (4) baseline DXA value, age and height.

|  |  | **Men** | | | | | | | | | | | |
| --- | --- | --- | --- | --- | --- | --- | --- | --- | --- | --- | --- | --- | --- |
|  |  | **Adjusted for baseline bone** | | | **Adjusted for baseline bone and age** | | | **Adjusted for baseline bone, age and weight** | | | **Adjusted for baseline bone, age and height** | | |
|  |  | **Beta** | **95% CI** | **p-value** | **Beta** | **95% CI** | **p-value** | **Beta** | **95% CI** | **p-value** | **Beta** | **95% CI** | **p-value** |
| **aBMD** | **B-CTx** | -0.07 | (-0.29,0.14) | 0.49 | -0.05 | (-0.25,0.16) | 0.66 | -0.04 | (-0.25,0.17) | 0.7 | -0.04 | (-0.25,0.16) | 0.67 |
|  | **PINP** | 0 | (-0.21,0.22) | 0.96 | -0.01 | (-0.21,0.20) | 0.95 | 0 | (-0.20,0.21) | 0.97 | -0.01 | (-0.22,0.20) | 0.93 |
|  | **PTH** | 0.08 | (-0.14,0.30) | 0.49 | 0.12 | (-0.10,0.34) | 0.27 | 0.1 | (-0.12,0.32) | 0.37 | 0.12 | (-0.10,0.34) | 0.27 |
|  | **25(OH)D** | **-0.24*** | **(-0.45,-0.03)** | **0.03** | **-0.25*** | **(-0.45,-0.04)** | **0.02** | **-0.22*** | **(-0.43,-0.01)** | **0.04** | **-0.24*** | **(-0.45,-0.03)** | **0.02** |
| **BMC** | **B-CTx** | -0.13 | (-0.38,0.13) | 0.33 | -0.08 | (-0.33,0.17) | 0.53 | -0.07 | (-0.32,0.18) | 0.56 | -0.08 | (-0.33,0.17) | 0.55 |
|  | **PINP** | -0.06 | (-0.32,0.19) | 0.63 | -0.07 | (-0.32,0.18) | 0.57 | -0.06 | (-0.31,0.18) | 0.61 | -0.08 | (-0.32,0.17) | 0.55 |
|  | **PTH** | 0.09 | (-0.18,0.36) | 0.53 | 0.15 | (-0.12,0.41) | 0.28 | 0.14 | (-0.13,0.41) | 0.32 | 0.14 | (-0.12,0.41) | 0.29 |
|  | **25(OH)D** | -0.09 | (-0.35,0.17) | 0.52 | -0.1 | (-0.35,0.15) | 0.44 | -0.08 | (-0.34,0.18) | 0.54 | -0.09 | (-0.35,0.16) | 0.47 |
| **BA** | **B-CTx** | -0.04 | (-0.21,0.13) | 0.66 | -0.02 | (-0.19,0.15) | 0.83 | -0.02 | (-0.20,0.15) | 0.82 | 0 | (-0.17,0.18) | 0.97 |
|  | **PINP** | -0.05 | (-0.22,0.12) | 0.57 | -0.05 | (-0.22,0.12) | 0.54 | -0.06 | (-0.23,0.12) | 0.53 | -0.05 | (-0.22,0.12) | 0.55 |
|  | **PTH** | -0.01 | (-0.20,0.17) | 0.88 | 0.01 | (-0.18,0.20) | 0.91 | 0.02 | (-0.17,0.21) | 0.87 | 0 | (-0.18,0.19) | 1 |
|  | **25(OH)D** | 0.15 | (-0.02,0.33) | 0.09 | 0.14 | (-0.03,0.32) | 0.1 | 0.15 | (-0.03,0.32) | 0.1 | 0.15 | (-0.02,0.32) | 0.09 |
|  |  | **Women** | | | | | | | | | | | |
|  |  | **Adjusted for baseline bone** | | | **Adjusted for baseline bone and age** | | | **Adjusted for baseline bone, age and weight** | | | **Adjusted for baseline bone, age and height** | | |
|  |  | **Beta** | **95% CI** | **p-value** | **Beta** | **95% CI** | **p-value** | **Beta** | **95% CI** | **p-value** | **Beta** | **95% CI** | **p-value** |
| **aBMD** | **B-CTx** | -0.16 | (-0.36,0.04) | 0.12 | -0.14 | (-0.34,0.06) | 0.18 | -0.11 | (-0.32,0.09) | 0.28 | -0.14 | (-0.34,0.06) | 0.17 |
|  | **PINP** | -0.06 | (-0.26,0.13) | 0.53 | -0.06 | (-0.25,0.14) | 0.57 | -0.05 | (-0.24,0.15) | 0.64 | -0.06 | (-0.25,0.13) | 0.54 |
|  | **PTH** | -0.02 | (-0.21,0.18) | 0.87 | 0.01 | (-0.19,0.20) | 0.92 | 0 | (-0.20,0.19) | 0.98 | 0.01 | (-0.19,0.20) | 0.93 |
|  | **25(OH)D** | -0.13 | (-0.32,0.06) | 0.17 | -0.13 | (-0.31,0.06) | 0.18 | -0.11 | (-0.29,0.08) | 0.26 | -0.15 | (-0.33,0.04) | 0.12 |
| **BMC** | **B-CTx** | -0.18 | (-0.47,0.10) | 0.21 | -0.17 | (-0.46,0.12) | 0.26 | -0.12 | (-0.41,0.17) | 0.41 | -0.19 | (-0.48,0.10) | 0.2 |
|  | **PINP** | -0.07 | (-0.35,0.21) | 0.62 | -0.06 | (-0.34,0.22) | 0.67 | -0.04 | (-0.31,0.24) | 0.8 | -0.07 | (-0.35,0.20) | 0.59 |
|  | **PTH** | -0.01 | (-0.29,0.26) | 0.93 | 0.01 | (-0.27,0.29) | 0.95 | -0.01 | (-0.29,0.27) | 0.95 | 0.01 | (-0.27,0.29) | 0.96 |
|  | **25(OH)D** | -0.21 | (-0.48,0.06) | 0.13 | -0.21 | (-0.48,0.06) | 0.13 | -0.16 | (-0.43,0.11) | 0.24 | -0.24 | (-0.51,0.03) | 0.08 |
| **BA** | **B-CTx** | 0 | (-0.17,0.16) | 0.96 | -0.03 | (-0.21,0.14) | 0.71 | 0 | (-0.18,0.18) | 0.98 | -0.03 | (-0.20,0.15) | 0.77 |
|  | **PINP** | 0.01 | (-0.16,0.17) | 0.93 | -0.01 | (-0.18,0.16) | 0.94 | 0.02 | (-0.15,0.19) | 0.82 | 0.01 | (-0.16,0.17) | 0.95 |
|  | **PTH** | 0.01 | (-0.15,0.18) | 0.86 | 0 | (-0.18,0.17) | 0.98 | -0.01 | (-0.18,0.17) | 0.94 | 0 | (-0.17,0.17) | 0.98 |
|  | **25(OH)D** | -0.08 | (-0.25,0.08) | 0.32 | -0.08 | (-0.24,0.09) | 0.35 | -0.06 | (-0.23,0.10) | 0.44 | -0.1 | (-0.26,0.07) | 0.25 |

Supplementary Table 2. Associations between baseline biomarkers (Fisher-Yates Z scores) and annualized yearly percentage change (calculated from the prospective measurements) in radius pQCT measures, in men and women adjusted for: (1) baseline pQCT value; (2) baseline pQCT value and age; (3) baseline pQCT value, age and weight; (4) baseline pQCT value, age and height.

|  |  | **Men** | | | | | | | | | | | | | |
| --- | --- | --- | --- | --- | --- | --- | --- | --- | --- | --- | --- | --- | --- | --- | --- |
|  |  | **Adjusted for baseline bone** | | | **Adjusted for baseline bone and age** | | | **Adjusted for baseline bone, age and weight** | | | **Adjusted for baseline bone, age and height** | | | |  |
|  |  | Beta | 95% CI | p-value | Beta | 95% CI | p-value | Beta | 95% CI | p-value | Beta | 95% CI | p-value |  |  |
| **Tr vBMD (4%)** | **B-CTx** | -0.15 | (-0.82,0.52) | 0.65 | -0.15 | (-0.83,0.52) | 0.65 | -0.13 | (-0.80,0.55) | 0.71 | -0.11 | (-0.79,0.57) | 0.74 |  |  |
|  | **PINP** | 0.31 | (-0.34,0.96) | 0.34 | 0.32 | (-0.33,0.97) | 0.34 | 0.35 | (-0.30,1.01) | 0.29 | 0.31 | (-0.34,0.97) | 0.34 |  |  |
|  | **PTH** | 0.26 | (-0.43,0.95) | 0.45 | 0.24 | (-0.46,0.94) | 0.5 | 0.25 | (-0.45,0.95) | 0.48 | 0.25 | (-0.46,0.95) | 0.49 |  |  |
|  | **25(OH)D** | -0.08 | (-0.71,0.55) | 0.8 | -0.08 | (-0.71,0.56) | 0.81 | -0.04 | (-0.68,0.60) | 0.91 | -0.07 | (-0.70,0.56) | 0.82 |  |  |
| **Tot vBMD (4%)** | **B-CTx** | 0.14 | (-0.52,0.80) | 0.67 | 0.12 | (-0.53,0.76) | 0.72 | 0.16 | (-0.49,0.80) | 0.63 | 0.12 | (-0.53,0.77) | 0.72 |  |  |
|  | **PINP** | -0.18 | (-0.81,0.45) | 0.58 | -0.26 | (-0.88,0.36) | 0.41 | -0.22 | (-0.84,0.40) | 0.49 | -0.26 | (-0.88,0.37) | 0.42 |  |  |
|  | **PTH** | -0.23 | (-0.91,0.45) | 0.5 | -0.15 | (-0.82,0.52) | 0.66 | -0.14 | (-0.81,0.53) | 0.69 | -0.15 | (-0.82,0.53) | 0.67 |  |  |
|  | **25(OH)D** | -0.21 | (-0.83,0.40) | 0.49 | -0.21 | (-0.81,0.39) | 0.5 | -0.16 | (-0.76,0.45) | 0.61 | -0.21 | (-0.81,0.40) | 0.5 |  |  |
| **CSA (4%)** | **B-CTx** | -0.13 | (-0.89,0.62) | 0.72 | -0.26 | (-1.01,0.49) | 0.5 | -0.26 | (-1.02,0.50) | 0.5 | -0.2 | (-0.96,0.56) | 0.6 |  |  |
|  | **PINP** | 0.33 | (-0.43,1.08) | 0.39 | 0.32 | (-0.43,1.06) | 0.4 | 0.32 | (-0.43,1.07) | 0.4 | 0.3 | (-0.44,1.05) | 0.43 |  |  |
|  | **PTH** | 0.31 | (-0.50,1.11) | 0.45 | 0.14 | (-0.68,0.95) | 0.74 | 0.13 | (-0.69,0.95) | 0.75 | 0.16 | (-0.65,0.98) | 0.69 |  |  |
|  | **25(OH)D** | -0.07 | (-0.81,0.67) | 0.85 | 0 | (-0.74,0.74) | 1 | 0 | (-0.75,0.74) | 1 | -0.01 | (-0.75,0.73) | 0.97 |  |  |
| **BSIc(4%)** | **B-CTx** | 0.21 | (-0.72,1.13) | 0.66 | 0.15 | (-0.76,1.07) | 0.75 | 0.17 | (-0.74,1.08) | 0.71 | 0.19 | (-0.72,1.11) | 0.68 |  |  |
|  | **PINP** | 0.1 | (-0.80,1.00) | 0.83 | -0.04 | (-0.94,0.86) | 0.93 | -0.01 | (-0.91,0.89) | 0.98 | -0.07 | (-0.97,0.83) | 0.88 |  |  |
|  | **PTH** | -0.35 | (-1.30,0.59) | 0.46 | -0.26 | (-1.21,0.69) | 0.59 | -0.26 | (-1.20,0.69) | 0.59 | -0.25 | (-1.19,0.70) | 0.61 |  |  |
|  | **25(OH)D** | -0.23 | (-1.08,0.62) | 0.6 | -0.26 | (-1.11,0.58) | 0.54 | -0.18 | (-1.03,0.68) | 0.68 | -0.25 | (-1.09,0.60) | 0.56 |  |  |
| **Ct. BMC (33%)** | **B-CTx** | -0.2 | (-0.49,0.10) | 0.19 | -0.2 | (-0.49,0.09) | 0.18 | -0.21 | (-0.50,0.08) | 0.16 | -0.2 | (-0.49,0.09) | 0.18 |  |  |
|  | **PINP** | **-0.33*** | **(-0.60,-0.06)** | **0.02** | **-0.37**** | **(-0.64,-0.10)** | **0.01** | **-0.37**** | **(-0.64,-0.10)** | **0.01** | **-0.38**** | **(-0.65,-0.10)** | **0.01** |  |  |
|  | **PTH** | 0.02 | (-0.27,0.31) | 0.9 | 0.09 | (-0.20,0.38) | 0.55 | 0.08 | (-0.21,0.37) | 0.57 | 0.09 | (-0.20,0.38) | 0.54 |  |  |
|  | **25(OH)D** | 0.09 | (-0.18,0.36) | 0.53 | 0.06 | (-0.21,0.33) | 0.66 | 0.08 | (-0.20,0.35) | 0.57 | 0.06 | (-0.21,0.33) | 0.67 |  |  |
| **Ct. vBMD (33%)** | **B-CTx** | -0.19 | (-0.40,0.03) | 0.09 | -0.19 | (-0.40,0.02) | 0.08 | -0.19 | (-0.40,0.02) | 0.08 | -0.19 | (-0.40,0.02) | 0.08 |  |  |
|  | **PINP** | **-0.25*** | **(-0.45,-0.06)** | **0.01** | **-0.28**** | **(-0.47,-0.08)** | **0.01** | **-0.28**** | **(-0.47,-0.08)** | **0.01** | **-0.27**** | **(-0.47,-0.08)** | **0.01** |  |  |
|  | **PTH** | -0.06 | (-0.28,0.15) | 0.58 | -0.02 | (-0.24,0.20) | 0.87 | -0.02 | (-0.24,0.20) | 0.87 | -0.02 | (-0.25,0.20) | 0.83 |  |  |
|  | **25(OH)D** | 0.03 | (-0.17,0.22) | 0.79 | 0 | (-0.19,0.19) | 1 | 0 | (-0.20,0.20) | 0.99 | -0.01 | (-0.20,0.19) | 0.96 |  |  |
| **Ct. CSA (33%)** | **B-CTx** | -0.17 | (-0.41,0.07) | 0.16 | -0.16 | (-0.40,0.07) | 0.17 | -0.17 | (-0.41,0.06) | 0.14 | -0.17 | (-0.41,0.06) | 0.15 |  |  |
|  | **PINP** | -0.17 | (-0.40,0.06) | 0.15 | -0.19 | (-0.42,0.04) | 0.1 | -0.2 | (-0.42,0.03) | 0.09 | -0.22 | (-0.46,0.01) | 0.06 |  |  |
|  | **PTH** | 0.02 | (-0.24,0.29) | 0.86 | 0.1 | (-0.17,0.36) | 0.47 | 0.08 | (-0.17,0.34) | 0.52 | 0.11 | (-0.15,0.37) | 0.41 |  |  |
|  | **25(OH)D** | 0.08 | (-0.14,0.31) | 0.47 | 0.06 | (-0.16,0.29) | 0.58 | 0.1 | (-0.12,0.33) | 0.37 | 0.08 | (-0.14,0.31) | 0.48 |  |  |
| **CSA (33%)** | **B-CTx** | 0.39 | (-0.07,0.85) | 0.1 | 0.41 | (-0.06,0.87) | 0.09 | 0.43 | (-0.03,0.89) | 0.07 | 0.43 | (-0.02,0.89) | 0.06 |  |  |
|  | **PINP** | **0.48*** | **(0.04,0.92)** | **0.03** | **0.48*** | **(0.04,0.92)** | **0.03** | **0.51*** | **(0.07,0.95)** | **0.02** | **0.45*** | **(0.01,0.88)** | **0.05** |  |  |
|  | **PTH** | 0.28 | (-0.20,0.75) | 0.25 | 0.35 | (-0.14,0.84) | 0.16 | 0.35 | (-0.14,0.84) | 0.16 | 0.39 | (-0.10,0.88) | 0.12 |  |  |
|  | **25(OH)D** | 0.12 | (-0.32,0.57) | 0.59 | 0.11 | (-0.34,0.55) | 0.64 | 0.16 | (-0.29,0.61) | 0.48 | 0.15 | (-0.30,0.59) | 0.51 |  |  |
|  |  | **Women** | | | | | | | | | | | | | |
|  |  | **Adjusted for baseline bone** | | | **Adjusted for baseline bone and age** | | | **Adjusted for baseline bone, age and weight** | | | **Adjusted for baseline bone, age and height** | | | |  |
|  |  | **Beta** | **95% CI** | **p-value** | **Beta** | **95% CI** | **p-value** | **Beta** | **95% CI** | **p-value** | **Beta** | **95% CI** | **p-value** |  |  |
| **Tr vBMD (4%)** | **B-CTx** | **-1.56***** | **(-2.41,-0.70)** | **<0.01** | **-1.25**** | **(-2.14,-0.36)** | **0.01** | **-1.25**** | **(-2.16,-0.34)** | **0.01** | **-1.25**** | **(-2.13,-0.36)** | **0.01** |  |  |
|  | **PINP** | **-1.09**** | **(-1.86,-0.33)** | **0.01** | **-0.90*** | **(-1.66,-0.14)** | **0.02** | **-0.89*** | **(-1.66,-0.12)** | **0.02** | **-0.89*** | **(-1.65,-0.12)** | **0.02** |  |  |
|  | **PTH** | 0.6 | (-0.24,1.43) | 0.16 | **0.99*** | **(0.16,1.81)** | **0.02** | **0.98*** | **(0.16,1.81)** | **0.02** | **1.03*** | **(0.20,1.85)** | **0.01** |  |  |
|  | **25(OH)D** | -0.04 | (-0.84,0.77) | 0.93 | -0.02 | (-0.80,0.76) | 0.96 | 0.01 | (-0.79,0.80) | 0.98 | -0.05 | (-0.84,0.73) | 0.89 |  |  |
| **Tot vBMD (4%)** | **B-CTx** | -0.46 | (-1.06,0.15) | 0.14 | -0.3 | (-0.92,0.33) | 0.35 | -0.29 | (-0.92,0.35) | 0.37 | -0.3 | (-0.92,0.33) | 0.35 |  |  |
|  | **PINP** | -0.41 | (-0.95,0.13) | 0.13 | -0.36 | (-0.89,0.18) | 0.19 | -0.35 | (-0.89,0.18) | 0.2 | -0.36 | (-0.89,0.18) | 0.19 |  |  |
|  | **PTH** | -0.05 | (-0.62,0.52) | 0.86 | 0.09 | (-0.49,0.66) | 0.76 | 0.08 | (-0.49,0.66) | 0.78 | 0.1 | (-0.48,0.67) | 0.74 |  |  |
|  | **25(OH)D** | -0.17 | (-0.70,0.37) | 0.54 | -0.15 | (-0.69,0.38) | 0.56 | -0.14 | (-0.68,0.40) | 0.6 | -0.16 | (-0.70,0.37) | 0.55 |  |  |
| **CSA (4%)** | **B-CTx** | 0.01 | (-0.66,0.67) | 0.98 | -0.09 | (-0.83,0.65) | 0.81 | -0.04 | (-0.79,0.72) | 0.92 | -0.1 | (-0.83,0.64) | 0.79 |  |  |
|  | **PINP** | 0.1 | (-0.51,0.71) | 0.76 | 0.05 | (-0.58,0.69) | 0.87 | 0.08 | (-0.56,0.72) | 0.8 | 0.08 | (-0.55,0.71) | 0.81 |  |  |
|  | **PTH** | 0.23 | (-0.42,0.89) | 0.48 | 0.19 | (-0.50,0.88) | 0.58 | 0.19 | (-0.50,0.88) | 0.59 | 0.26 | (-0.43,0.95) | 0.46 |  |  |
|  | **25(OH)D** | 0.22 | (-0.42,0.87) | 0.49 | 0.22 | (-0.42,0.87) | 0.49 | 0.27 | (-0.38,0.92) | 0.42 | 0.15 | (-0.49,0.80) | 0.64 |  |  |
| **BSIc(4%)** | **B-CTx** | **-0.94*** | **(-1.80,-0.08)** | **0.03** | -0.77 | (-1.65,0.11) | 0.08 | -0.76 | (-1.64,0.13) | 0.09 | -0.8 | (-1.68,0.08) | 0.07 |  |  |
|  | **PINP** | -0.58 | (-1.36,0.19) | 0.14 | -0.53 | (-1.30,0.24) | 0.18 | -0.53 | (-1.30,0.25) | 0.18 | -0.54 | (-1.31,0.23) | 0.17 |  |  |
|  | **PTH** | 0.19 | (-0.60,0.99) | 0.63 | 0.4 | (-0.40,1.21) | 0.33 | 0.39 | (-0.42,1.20) | 0.34 | 0.43 | (-0.38,1.24) | 0.29 |  |  |
|  | **25(OH)D** | -0.04 | (-0.79,0.71) | 0.91 | -0.07 | (-0.81,0.67) | 0.86 | -0.04 | (-0.79,0.71) | 0.92 | -0.1 | (-0.84,0.65) | 0.79 |  |  |
| **Ct. BMC (33%)** | **B-CTx** | 0.01 | (-0.49,0.51) | 0.97 | 0.05 | (-0.46,0.56) | 0.85 | 0.05 | (-0.46,0.56) | 0.85 | 0.03 | (-0.49,0.54) | 0.92 |  |  |
|  | **PINP** | 0.29 | (-0.15,0.72) | 0.19 | 0.29 | (-0.15,0.72) | 0.19 | 0.29 | (-0.15,0.73) | 0.19 | 0.28 | (-0.16,0.72) | 0.21 |  |  |
|  | **PTH** | 0.1 | (-0.31,0.51) | 0.63 | 0.16 | (-0.27,0.59) | 0.47 | 0.16 | (-0.27,0.59) | 0.47 | 0.18 | (-0.26,0.61) | 0.42 |  |  |
|  | **25(OH)D** | 0.07 | (-0.34,0.47) | 0.75 | 0.06 | (-0.35,0.47) | 0.77 | 0.06 | (-0.35,0.47) | 0.77 | 0.05 | (-0.36,0.46) | 0.82 |  |  |
| **Ct. vBMD (33%)** | **B-CTx** | -0.17 | (-0.43,0.10) | 0.22 | -0.14 | (-0.40,0.12) | 0.29 | -0.14 | (-0.41,0.12) | 0.29 | -0.14 | (-0.40,0.12) | 0.27 |  |  |
|  | **PINP** | -0.11 | (-0.34,0.11) | 0.32 | -0.14 | (-0.36,0.08) | 0.21 | -0.14 | (-0.36,0.08) | 0.21 | -0.14 | (-0.36,0.08) | 0.22 |  |  |
|  | **PTH** | -0.01 | (-0.22,0.20) | 0.94 | 0.06 | (-0.15,0.27) | 0.58 | 0.06 | (-0.15,0.27) | 0.58 | 0.08 | (-0.13,0.29) | 0.48 |  |  |
|  | **25(OH)D** | -0.01 | (-0.21,0.19) | 0.93 | -0.03 | (-0.23,0.17) | 0.74 | -0.03 | (-0.24,0.17) | 0.74 | -0.06 | (-0.26,0.14) | 0.58 |  |  |
| **Ct. CSA (33%)** | **B-CTx** | -0.22 | (-0.65,0.22) | 0.33 | -0.13 | (-0.58,0.32) | 0.57 | -0.13 | (-0.58,0.32) | 0.58 | -0.14 | (-0.59,0.31) | 0.54 |  |  |
|  | **PINP** | 0.07 | (-0.32,0.46) | 0.72 | 0.1 | (-0.29,0.48) | 0.62 | 0.09 | (-0.30,0.48) | 0.65 | 0.09 | (-0.30,0.48) | 0.64 |  |  |
|  | **PTH** | 0.04 | (-0.33,0.41) | 0.83 | 0.15 | (-0.24,0.53) | 0.45 | 0.15 | (-0.24,0.54) | 0.44 | 0.16 | (-0.23,0.55) | 0.43 |  |  |
|  | **25(OH)D** | 0.14 | (-0.23,0.51) | 0.45 | 0.13 | (-0.23,0.50) | 0.47 | 0.15 | (-0.22,0.51) | 0.44 | 0.13 | (-0.24,0.50) | 0.49 |  |  |
| **CSA (33%)** | **B-CTx** | 0.09 | (-0.36,0.54) | 0.69 | 0.07 | (-0.43,0.57) | 0.78 | 0.12 | (-0.39,0.64) | 0.63 | 0.07 | (-0.43,0.57) | 0.78 |  |  |
|  | **PINP** | -0.12 | (-0.53,0.29) | 0.56 | -0.13 | (-0.56,0.30) | 0.55 | -0.15 | (-0.57,0.28) | 0.5 | -0.15 | (-0.57,0.28) | 0.50 |  |  |
|  | **PTH** | 0.39 | (-0.05,0.82) | 0.08 | 0.4 | (-0.06,0.85) | 0.09 | 0.4 | (-0.05,0.86) | 0.08 | 0.44 | (-0.01,0.90) | 0.06 |  |  |
|  | **25(OH)D** | 0 | (-0.43,0.43) | 1 | 0.01 | (-0.43,0.44) | 0.98 | 0.03 | (-0.41,0.47) | 0.9 | -0.02 | (-0.46,0.42) | 0.92 |  |  |

Supplementary Table 3. Associations between baseline biomarkers (Fisher-Yates Z scores) and annualized yearly percentage change (calculated from the prospective measurements) in tibia pQCT measures, in men and women adjusted for: (1) baseline pQCT value; (2) baseline pQCT value and age; (3) baseline pQCT value, age and weight; (4) baseline pQCT value, age and height.

|  |  | **Men** | | | | | | | | | | | |
| --- | --- | --- | --- | --- | --- | --- | --- | --- | --- | --- | --- | --- | --- |
|  |  | **Adjusted for baseline bone** | | | **Adjusted for baseline bone and age** | | | **Adjusted for baseline bone, age and weight** | | | **Adjusted for baseline bone, age and height** | | |
|  |  | **Beta** | **95% CI** | **p-value** | **Beta** | **95% CI** | **p-value** | **Beta** | **95% CI** | **p-value** | **Beta** | **95% CI** | **p-value** |
| **Tr vBMD (4%)** | **B-CTx** | 0.02 | (-0.37,0.42) | 0.9 | 0.03 | (-0.37,0.42) | 0.9 | 0.03 | (-0.37,0.43) | 0.88 | 0.05 | (-0.35,0.45) | 0.79 |
|  | **PINP** | -0.13 | (-0.52,0.26) | 0.5 | -0.15 | (-0.55,0.24) | 0.45 | -0.14 | (-0.53,0.26) | 0.49 | -0.16 | (-0.56,0.23) | 0.42 |
|  | **PTH** | 0.24 | (-0.14,0.63) | 0.21 | 0.25 | (-0.14,0.65) | 0.2 | 0.24 | (-0.16,0.63) | 0.24 | 0.26 | (-0.14,0.65) | 0.2 |
|  | **25(OH)D** | -0.27 | (-0.66,0.11) | 0.17 | -0.27 | (-0.66,0.12) | 0.17 | -0.22 | (-0.62,0.18) | 0.27 | -0.26 | (-0.65,0.13) | 0.18 |
| **Tot vBMD (4%)** | **B-CTx** | -0.22 | (-0.54,0.11) | 0.19 | -0.22 | (-0.54,0.11) | 0.18 | -0.2 | (-0.52,0.11) | 0.2 | -0.21 | (-0.53,0.12) | 0.21 |
|  | **PINP** | -0.23 | (-0.54,0.09) | 0.16 | -0.26 | (-0.58,0.06) | 0.11 | -0.23 | (-0.54,0.08) | 0.15 | -0.27 | (-0.59,0.05) | 0.1 |
|  | **PTH** | 0.50** | (0.18,0.83) | <0.01 | 0.53** | (0.20,0.86) | <0.01 | 0.50** | (0.18,0.82) | <0.01 | 0.54** | (0.21,0.86) | <0.01 |
|  | **25(OH)D** | -0.08 | (-0.41,0.24) | 0.61 | -0.08 | (-0.41,0.24) | 0.61 | 0.02 | (-0.31,0.34) | 0.92 | -0.08 | (-0.40,0.25) | 0.65 |
| **CSA (4%)** | **B-CTx** | 0.18 | (-0.12,0.48) | 0.24 | 0.19 | (-0.11,0.49) | 0.2 | 0.19 | (-0.11,0.49) | 0.22 | 0.19 | (-0.11,0.50) | 0.21 |
|  | **PINP** | 0.16 | (-0.14,0.45) | 0.3 | 0.15 | (-0.15,0.45) | 0.33 | 0.14 | (-0.16,0.45) | 0.36 | 0.15 | (-0.15,0.45) | 0.32 |
|  | **PTH** | -0.44** | (-0.75,-0.14) | <0.01 | -0.43** | (-0.74,-0.12) | 0.01 | -0.43** | (-0.74,-0.12) | 0.01 | -0.45** | (-0.77,-0.14) | 0.01 |
|  | **25(OH)D** | 0.02 | (-0.29,0.32) | 0.92 | 0.01 | (-0.30,0.31) | 0.96 | 0.01 | (-0.31,0.31) | 0.99 | 0.01 | (-0.30,0.32) | 0.97 |
| **BSIc(4%)** | **B-CTx** | -0.19 | (-0.78,0.40) | 0.52 | -0.2 | (-0.78,0.39) | 0.51 | -0.23 | (-0.80,0.33) | 0.42 | -0.17 | (-0.76,0.41) | 0.56 |
|  | **PINP** | -0.39 | (-0.96,0.18) | 0.18 | -0.45 | (-1.03,0.12) | 0.12 | -0.45 | (-1.01,0.10) | 0.11 | -0.49 | (-1.06,0.09) | 0.1 |
|  | **PTH** | 0.86** | (0.29,1.43) | <0.01 | 0.92** | (0.35,1.49) | <0.01 | 0.86** | (0.30,1.42) | <0.01 | 0.94** | (0.36,1.51) | <0.01 |
|  | **25(OH)D** | -0.24 | (-0.82,0.33) | 0.4 | -0.25 | (-0.82,0.32) | 0.39 | -0.07 | (-0.64,0.50) | 0.8 | -0.24 | (-0.81,0.33) | 0.41 |
| **Ct. BMC (38%)** | **B-CTx** | -0.08 | (-0.20,0.03) | 0.16 | -0.08 | (-0.20,0.03) | 0.15 | -0.08 | (-0.20,0.03) | 0.16 | -0.08 | (-0.20,0.03) | 0.16 |
|  | **PINP** | -0.06 | (-0.18,0.05) | 0.29 | -0.07 | (-0.19,0.04) | 0.22 | -0.07 | (-0.19,0.04) | 0.23 | -0.08 | (-0.19,0.04) | 0.2 |
|  | **PTH** | 0.06 | (-0.06,0.18) | 0.32 | 0.07 | (-0.05,0.19) | 0.23 | 0.07 | (-0.05,0.19) | 0.23 | 0.07 | (-0.04,0.19) | 0.22 |
|  | **25(OH)D** | -0.01 | (-0.13,0.11) | 0.87 | -0.01 | (-0.13,0.10) | 0.84 | -0.01 | (-0.13,0.12) | 0.93 | -0.01 | (-0.13,0.11) | 0.85 |
| **Ct. vBMD (38%)** | **B-CTx** | 0.01 | (-0.05,0.07) | 0.78 | 0.01 | (-0.05,0.07) | 0.79 | 0.01 | (-0.05,0.07) | 0.79 | 0.01 | (-0.06,0.07) | 0.83 |
|  | **PINP** | 0.01 | (-0.05,0.08) | 0.66 | 0.02 | (-0.05,0.08) | 0.64 | 0.02 | (-0.05,0.08) | 0.64 | 0.01 | (-0.05,0.08) | 0.66 |
|  | **PTH** | 0.01 | (-0.07,0.06) | 0.89 | -0.01 | (-0.07,0.06) | 0.79 | -0.01 | (-0.08,0.06) | 0.79 | -0.01 | (-0.08,0.06) | 0.75 |
|  | **25(OH)D** | -0.04 | (-0.10,0.02) | 0.18 | -0.04 | (-0.10,0.02) | 0.19 | -0.04 | (-0.11,0.02) | 0.18 | -0.04 | (-0.10,0.02) | 0.19 |
| **Ct. CSA (38%)** | **B-CTx** | -0.11 | (-0.24,0.01) | 0.07 | -0.11 | (-0.24,0.01) | 0.07 | -0.11 | (-0.24,0.01) | 0.08 | -0.11 | (-0.24,0.01) | 0.07 |
|  | **PINP** | -0.1 | (-0.22,0.03) | 0.12 | -0.11 | (-0.23,0.01) | 0.08 | -0.11 | (-0.23,0.02) | 0.09 | -0.12 | (-0.24,0.01) | 0.06 |
|  | **PTH** | 0.04 | (-0.08,0.17) | 0.49 | 0.06 | (-0.06,0.19) | 0.32 | 0.06 | (-0.06,0.19) | 0.33 | 0.07 | (-0.06,0.20) | 0.29 |
|  | **25(OH)D** | 0.04 | (-0.09,0.16) | 0.57 | 0.03 | (-0.09,0.16) | 0.61 | 0.05 | (-0.08,0.19) | 0.41 | 0.03 | (-0.09,0.16) | 0.59 |
| **CSA (38%)** | **B-CTx** | 0.06 | (-0.03,0.14) | 0.2 | 0.06 | (-0.03,0.15) | 0.17 | 0.05 | (-0.03,0.14) | 0.22 | 0.05 | (-0.03,0.14) | 0.2 |
|  | **PINP** | 0.04 | (-0.05,0.13) | 0.36 | 0.04 | (-0.05,0.12) | 0.38 | 0.03 | (-0.06,0.11) | 0.54 | 0.04 | (-0.04,0.12) | 0.35 |
|  | **PTH** | 0.05 | (-0.04,0.14) | 0.28 | 0.06 | (-0.02,0.15) | 0.15 | 0.06 | (-0.03,0.15) | 0.18 | 0.05 | (-0.04,0.14) | 0.25 |
|  | **25(OH)D** | 0.04 | (-0.05,0.13) | 0.4 | 0.03 | (-0.05,0.12) | 0.45 | 0.02 | (-0.07,0.11) | 0.64 | 0.03 | (-0.05,0.12) | 0.43 |
|  |  | **Women** | | | | | | | | | | | |
|  |  | **Adjusted for baseline bone** | | | **Adjusted for baseline bone and age** | | | **Adjusted for baseline bone, age and weight** | | | **Adjusted for baseline bone, age and height** | | |
|  |  | **Beta** | **95% CI** | **p-value** | **Beta** | **95% CI** | **p-value** | **Beta** | **95% CI** | **p-value** | **Beta** | **95% CI** | **p-value** |
| **Tr vBMD (4%)** | **B-CTx** | -0.03 | (-0.54,0.47) | 0.89 | 0.04 | (-0.48,0.56) | 0.88 | 0.01 | (-0.51,0.54) | 0.96 | 0.04 | (-0.48,0.56) | 0.87 |
|  | **PINP** | -0.03 | (-0.50,0.44) | 0.91 | 0.01 | (-0.47,0.48) | 0.98 | 0.01 | (-0.47,0.48) | 1 | 0.01 | (-0.47,0.48) | 0.99 |
|  | **PTH** | -0.02 | (-0.52,0.48) | 0.94 | 0.06 | (-0.46,0.59) | 0.81 | 0.04 | (-0.49,0.57) | 0.88 | 0.1 | (-0.42,0.63) | 0.7 |
|  | **25(OH)D** | 0.50* | (0.04,0.96) | 0.04 | 0.50* | (0.04,0.96) | 0.03 | 0.49* | (0.03,0.96) | 0.04 | 0.47* | (0.00,0.94) | 0.05 |
| **Tot vBMD (4%)** | **B-CTx** | 0.08 | (-0.29,0.45) | 0.67 | 0.11 | (-0.26,0.49) | 0.55 | 0.1 | (-0.28,0.48) | 0.61 | 0.12 | (-0.26,0.49) | 0.54 |
|  | **PINP** | 0.17 | (-0.17,0.51) | 0.33 | 0.18 | (-0.16,0.52) | 0.31 | 0.17 | (-0.17,0.52) | 0.31 | 0.18 | (-0.17,0.52) | 0.31 |
|  | **PTH** | 0.04 | (-0.32,0.40) | 0.83 | 0.09 | (-0.29,0.46) | 0.65 | 0.07 | (-0.31,0.45) | 0.71 | 0.1 | (-0.28,0.47) | 0.62 |
|  | **25(OH)D** | 0.01 | (-0.33,0.34) | 0.96 | 0.01 | (-0.33,0.35) | 0.96 | 0.01 | (-0.33,0.34) | 0.98 | 0.01 | (-0.34,0.34) | 1 |
| **CSA (4%)** | **B-CTx** | -0.03 | (-0.29,0.24) | 0.85 | -0.04 | (-0.32,0.24) | 0.78 | -0.03 | (-0.32,0.26) | 0.84 | -0.03 | (-0.31,0.25) | 0.82 |
|  | **PINP** | -0.09 | (-0.34,0.16) | 0.48 | -0.1 | (-0.36,0.16) | 0.45 | -0.09 | (-0.36,0.17) | 0.48 | -0.09 | (-0.35,0.17) | 0.51 |
|  | **PTH** | -0.1 | (-0.36,0.17) | 0.47 | -0.12 | (-0.41,0.17) | 0.41 | -0.12 | (-0.41,0.17) | 0.43 | -0.1 | (-0.39,0.19) | 0.49 |
|  | **25(OH)D** | 0.25* | (0.00,0.50) | 0.05 | 0.26* | (0.00,0.51) | 0.05 | 0.26* | (0.00,0.51) | 0.05 | 0.23 | (-0.03,0.49) | 0.09 |
| **BSIc(4%)** | **B-CTx** | 0.25 | (-0.47,0.97) | 0.49 | 0.33 | (-0.40,1.05) | 0.38 | 0.31 | (-0.43,1.04) | 0.41 | 0.31 | (-0.41,1.03) | 0.4 |
|  | **PINP** | 0.19 | (-0.48,0.85) | 0.58 | 0.21 | (-0.45,0.88) | 0.53 | 0.22 | (-0.45,0.88) | 0.52 | 0.19 | (-0.47,0.86) | 0.56 |
|  | **PTH** | 0.17 | (-0.52,0.85) | 0.63 | 0.26 | (-0.44,0.96) | 0.46 | 0.23 | (-0.47,0.94) | 0.51 | 0.3 | (-0.40,1.00) | 0.4 |
|  | **25(OH)D** | 0.27 | (-0.38,0.93) | 0.41 | 0.26 | (-0.39,0.92) | 0.43 | 0.26 | (-0.40,0.92) | 0.44 | 0.18 | (-0.49,0.84) | 0.6 |
| **Ct. BMC (38%)** | **B-CTx** | -0.14 | (-0.39,0.11) | 0.27 | -0.04 | (-0.28,0.21) | 0.75 | -0.03 | (-0.28,0.21) | 0.79 | -0.01 | (-0.26,0.23) | 0.91 |
|  | **PINP** | -0.13 | (-0.36,0.09) | 0.25 | -0.1 | (-0.32,0.11) | 0.35 | -0.08 | (-0.31,0.15) | 0.49 | -0.07 | (-0.29,0.15) | 0.51 |
|  | **PTH** | -0.09 | (-0.33,0.14) | 0.43 | 0.04 | (-0.20,0.28) | 0.74 | 0.04 | (-0.21,0.28) | 0.77 | 0.01 | (-0.23,0.25) | 0.96 |
|  | **25(OH)D** | -0.02 | (-0.26,0.22) | 0.86 | -0.02 | (-0.25,0.21) | 0.87 | -0.04 | (-0.27,0.19) | 0.74 | 0 | (-0.23,0.22) | 0.97 |
| **Ct. vBMD (38%)** | **B-CTx** | -0.06 | (-0.18,0.06) | 0.36 | -0.06 | (-0.18,0.06) | 0.32 | -0.07 | (-0.19,0.05) | 0.25 | -0.06 | (-0.18,0.06) | 0.33 |
|  | **PINP** | -0.1 | (-0.22,0.01) | 0.08 | -0.1 | (-0.21,0.02) | 0.1 | -0.08 | (-0.20,0.03) | 0.15 | -0.1 | (-0.21,0.02) | 0.1 |
|  | **PTH** | 0 | (-0.10,0.10) | 0.96 | -0.01 | (-0.12,0.10) | 0.86 | -0.02 | (-0.12,0.09) | 0.76 | -0.02 | (-0.13,0.09) | 0.72 |
|  | **25(OH)D** | -0.03 | (-0.13,0.08) | 0.62 | -0.03 | (-0.13,0.08) | 0.63 | -0.03 | (-0.13,0.07) | 0.62 | -0.02 | (-0.12,0.08) | 0.71 |
| **Ct. CSA (38%)** | **B-CTx** | -0.12 | (-0.35,0.11) | 0.3 | -0.01 | (-0.23,0.22) | 0.96 | -0.01 | (-0.24,0.22) | 0.96 | 0.02 | (-0.21,0.24) | 0.87 |
|  | **PINP** | -0.06 | (-0.27,0.15) | 0.57 | -0.01 | (-0.21,0.19) | 0.9 | -0.01 | (-0.22,0.20) | 0.93 | 0.02 | (-0.18,0.22) | 0.83 |
|  | **PTH** | -0.09 | (-0.31,0.14) | 0.45 | 0.05 | (-0.18,0.28) | 0.66 | 0.05 | (-0.18,0.28) | 0.66 | 0.01 | (-0.21,0.24) | 0.91 |
|  | **25(OH)D** | 0 | (-0.23,0.22) | 0.97 | -0.01 | (-0.22,0.21) | 0.93 | -0.01 | (-0.23,0.21) | 0.91 | 0.01 | (-0.20,0.22) | 0.94 |
| **CSA (38%)** | **B-CTx** | 0.17*** | (0.07,0.26) | <0.01 | 0.19*** | (0.09,0.29) | <0.01 | 0.18*** | (0.08,0.28) | <0.01 | 0.19*** | (0.09,0.29) | <0.01 |
|  | **PINP** | 0.11* | (0.02,0.20) | 0.01 | 0.12* | (0.03,0.21) | 0.01 | 0.11* | (0.02,0.20) | 0.02 | 0.11* | (0.02,0.21) | 0.02 |
|  | **PTH** | 0.11* | (0.01,0.21) | 0.02 | 0.13* | (0.02,0.23) | 0.02 | 0.12* | (0.01,0.22) | 0.03 | 0.12* | (0.01,0.23) | 0.03 |
|  | **25(OH)D** | 0.03 | (-0.06,0.13) | 0.51 | 0.03 | (-0.07,0.13) | 0.5 | 0.04 | (-0.06,0.13) | 0.47 | 0.04 | (-0.06,0.14) | 0.4 |
